# Nanobiosensors for revolutionizing parasitic infections diagnosis: a critical review to improve global health with an update on future challenges prospect

**DOI:** 10.1186/s40001-025-02685-2

**Published:** 2025-06-16

**Authors:** Soheil Sadr, Ashkan Hajjafari, Alireza Sazmand, Cinzia Santucciu, Giovanna Masala, Mahdi Soroushianfar, Shakiba Nazemian, Abbas Rahdar, Sadanand Pandey, Moez Guettari, Hassan Borji

**Affiliations:** 1 Department of Pathobiology, Faculty of Veterinary Medicine, Ferdowsi University of Mashhad, Mashhad, Iran; 2 Department of Pathobiology, Faculty of Veterinary Medicine Science, Science and Research Branch, Islamic Azad University, Tehran, Iran; 3 Department of Pathobiology, Faculty of Veterinary Medicine, Bu-Ali Sina University, Hamedan, 6517658978 Iran; 4 WOAH and NRL for Echinococcosis, Animal Health, Istituto Zooprofilattico Sperimentale Della Sardegna, 07100 Sassari, Italy; 5 Faculty of Veterinary Medicine, Ferdowsi University of Mashhad, Mashhad, Iran; 6 Department of Physics, University of Zabol, Zabol, Iran; 7 School of Bioengineering and Food Technology, Faculty of Applied Sciences and Biotechnology, Shoolini University, Solan, 173229 Himachal Pradesh India; 8 Department of Chemistry, College of Natural Science, Yeungnam University, 280 Daehak‐Ro, Gyeongsan, 38541 Korea; 9 Preparatory Institute for Engineering Studies of Tunis, Materials and Fluids, Laboratory LR19ES03, University of Tunis, Tunis, Tunisia

**Keywords:** Biomarkers, Helminth, Nanobiosensors, Nanotechnology, Parasites, Protozoa

## Abstract

Parasitic infections remain a serious public health issue globally, requiring prompt and precise diagnosis. Traditional diagnostic techniques, such as microscopic examinations, immunological methods, such as enzyme-linked immunosorbent assay (ELISA), and molecular tests, such as polymerase chain reaction (PCR), are standard tools for parasite identification. However, traditional methods are time-consuming and have less sensitivity and specificity than nanobiosensors. Hence, the current review aims to analyze the nanobiosensors in detecting globally important human parasites, *i.e.,* *Plasmodium*, *Leishmania*, *Echinococcus*, *Schistosoma*, and *Taenia*, emphasizing their significance in the early detection and analyzing their future challenges. Nanobiosensors provide efficient, sensitive, and rapid diagnosis of parasites’ antigens or genetic material using nanomaterials, such as nanowires, quantum dots (QDs), metallic nanoparticles, and carbon nanotubes, as well as identification of biomarkers, including excretory–secretory products and microRNAs. Nanobiosensors can utilize diverse nanomaterials such as gold nanoparticles (AuNPs) for the detection of *Plasmodium falciparum* histidine-rich protein 2 (*Pf*HRP2) in *Plasmodium*, carbon nanotubes (CNTs) functionalized with anti-EgAgB antibodies for *Echinococcus*, and QDs labeled with DNA probes for the detection of *Leishmania* kDNA. Regarding *Schistosoma*, graphene oxide (GO)-based nanobiosensors with a soluble egg antigen (SEA) binding, and for *Taenia*, metallic nanobiosensors can detect parasites’ biomarkers even at low concentrations. Challenges for using nanobiosensors in parasitic infection diagnosis include limitations in mass production, biological matrix interference, and the need for standardization. Development of multiplex nanobiosensors using polymer nanofibers or hybrid nanoparticles for simultaneous detection of multiple pathogens, along with integration of lab-on-a-chip technology for point-of-care (PoC) platforms, is an important future prospect that needs to be worked on. In conclusion, considering the rapidly ongoing advancement of nanobiosensors, it is expected that they will aid the detection, treatment, and management of parasitic infections by providing new avenues for early detection, improved treatment, and improved disease management in the future.

**Graphical Abstract:**

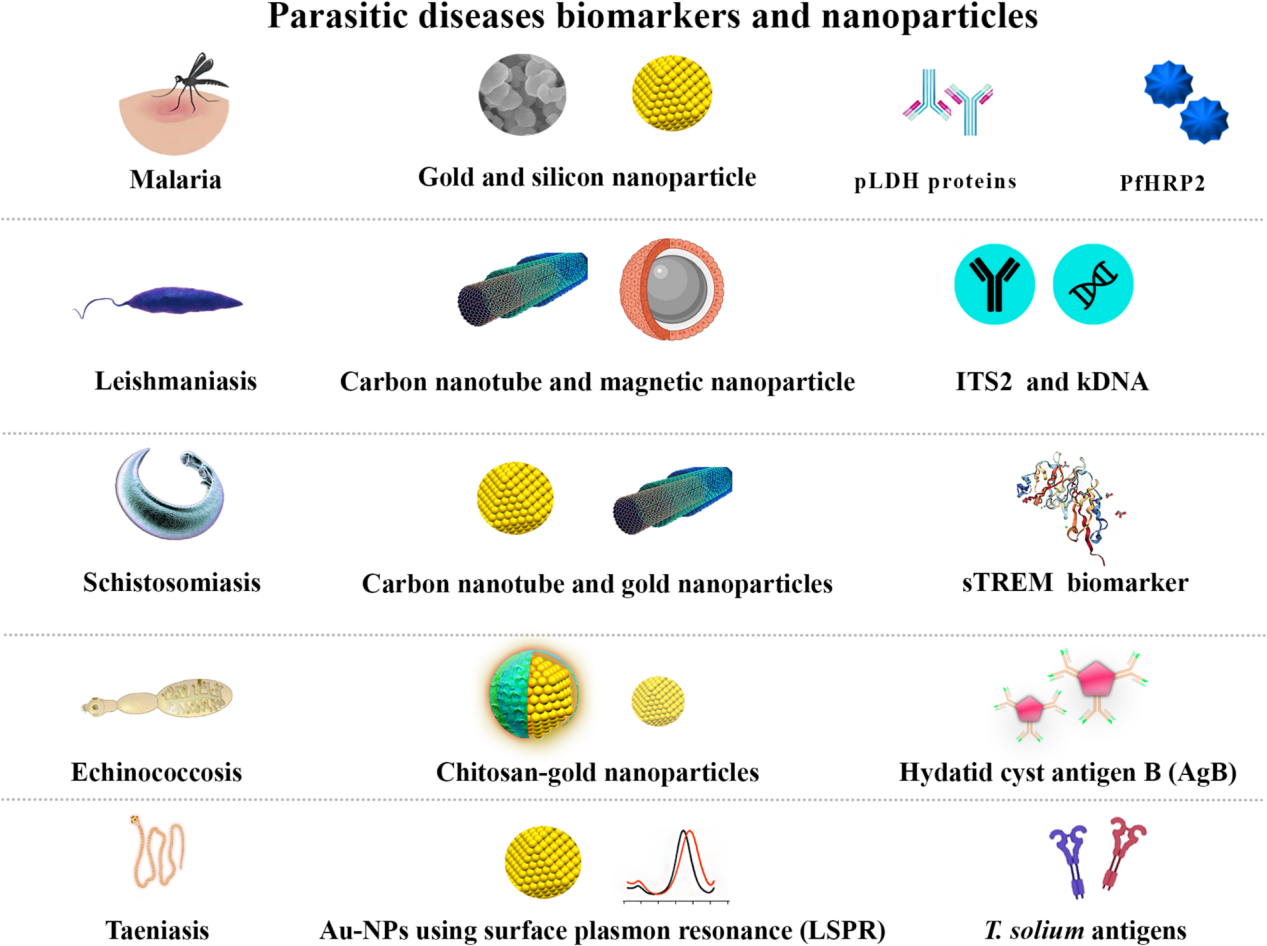

## Highlights

1. Helminth and protozoan infections remain a substantial public health issue
2. Nanobiosensors are highly sensitive for diagnosing zoonotic parasites
3. Nanobiosensors can detect parasites’ biomarkers, including ESPs and miRNAs
4. By overcoming the challenges, nanobiosensors could revolutionize the management of zoonotic parasites

## Introduction

Early diagnosis of parasitic infections is crucial for efficiently managing the disease and mitigating potential consequences [1–3]. However, conventional diagnostic techniques are constrained by their low sensitivity, limited specificity, and restricted accessibility [4–6]. For instance, microscopy as a golden standard has to be carried out only by an expert operator [7], since several parasite eggs cannot be distinguished morphologically [8, 9]. Moreover, serological assays such as immunohistochemistry (IHC), enzyme-linked immunosorbent assay (ELISA), immunoblotting (IB), and tests that detect the antigenic molecules of the parasites or the antibodies of the host [10, 11] are often hindered by cross-reactions or low sensitivity [12, 13].

Although molecular techniques have several advantages over traditional methods, they also have their own limitations [14, 15]. Collecting fresh specimens is necessary for molecular techniques, but can be difficult in remote or resource-limited areas [16, 17]. In addition, molecular techniques require special equipment [18, 19]. Moreover, traditional methods are time-consuming and have less sensitivity and specificity than nanobiosensors [20–22]. Nanobiosensors have several advantages in detecting parasitic infections, such as rapid, accurate, and cost-effective results [23–26]. Nanobiosensors can also provide more reliable diagnostic options for patients with parasitic diseases [27–30].Quantum dots (QDs) [31–34], nanowires [35], and carbon nanotubes [36, 37] can be used in the structure of a nanobiosensor and detect the parasite's biomarkers [36]. Nanobiosensors have a great sensitivity and specificity in identifying pathogens, encompassing the efficient and vulnerable detection of helminth antigens or genetic material [32, 38]. Furthermore, the enhancement of biosensing platforms enables the integration of  point of care (PoC), facilitating the timely identification of medical conditions and the commencement of appropriate treatment interventions [39, 40].

Nanobiosensors are a promising option for quickly and accurately detecting parasites, antigens, or genetic material, which is crucial for preventing zoonotic infections transmission and reducing health hazards [41–44]. As a result, its implementation has the potential to enable prompt diagnosis and efficient parasite infection management techniques [45–47]. Hence, the present article aims to comprehensively review the capabilities of nanobiosensors in transforming the detection and control of zoonotic parasitic infections, a significant public health issue. The present review discusses the performance of nanobiosensors for major human zoonotic parasites, including protozoan parasites such as *Plasmodium* and *Leishmania* spp. the causative agents of malaria and leishmaniasis; tapeworms such as *Taenia* spp, the causative agent of taeniasis, *Echinococcus granulosus* sensu lato (s.l.), agent of cystic echinococcosis (CE), and *Schistosoma mansoni, Schistosoma japonicum,* and *Schistosoma haematobium*, which are responsible for schistosomiasis.

## Nanobiosensors

Nanobiosensors are analytical and diagnostic tools that integrate nanotechnology with biology to identify and examine biological and chemical targets at the nanoscale [48]. Various techniques have been developed in recent years to fabricate and develop nanobiosensors [49–52]. Many nanomaterials are used for this process, including metallic nanoparticles, nanowires, and carbon nanotubes [53]. Nanoparticles with distinctive physical and chemical characteristics improve the sensitivity and specificity of nanobiosensors [54]. Nanomaterials may even be coated with biological molecules such as antibodies, enzymes, or DNA strands to engage with a specific analyte, which is a good way to ensure that the nanomaterials work in the desired manner [55, 56]. In addition, complex manufacturing methods such as electron beam writing, lithography, or self-assembly are used to accurately design nanobiosensors, enabling precise detection of analytes [57–60]. Furthermore, microfluidics and lab-on-a-chip (LoC) technologies can enhance the efficacy and reduce the size, allowing for quick and multiplexed analysis [40, 61].

**Fig. 1:**
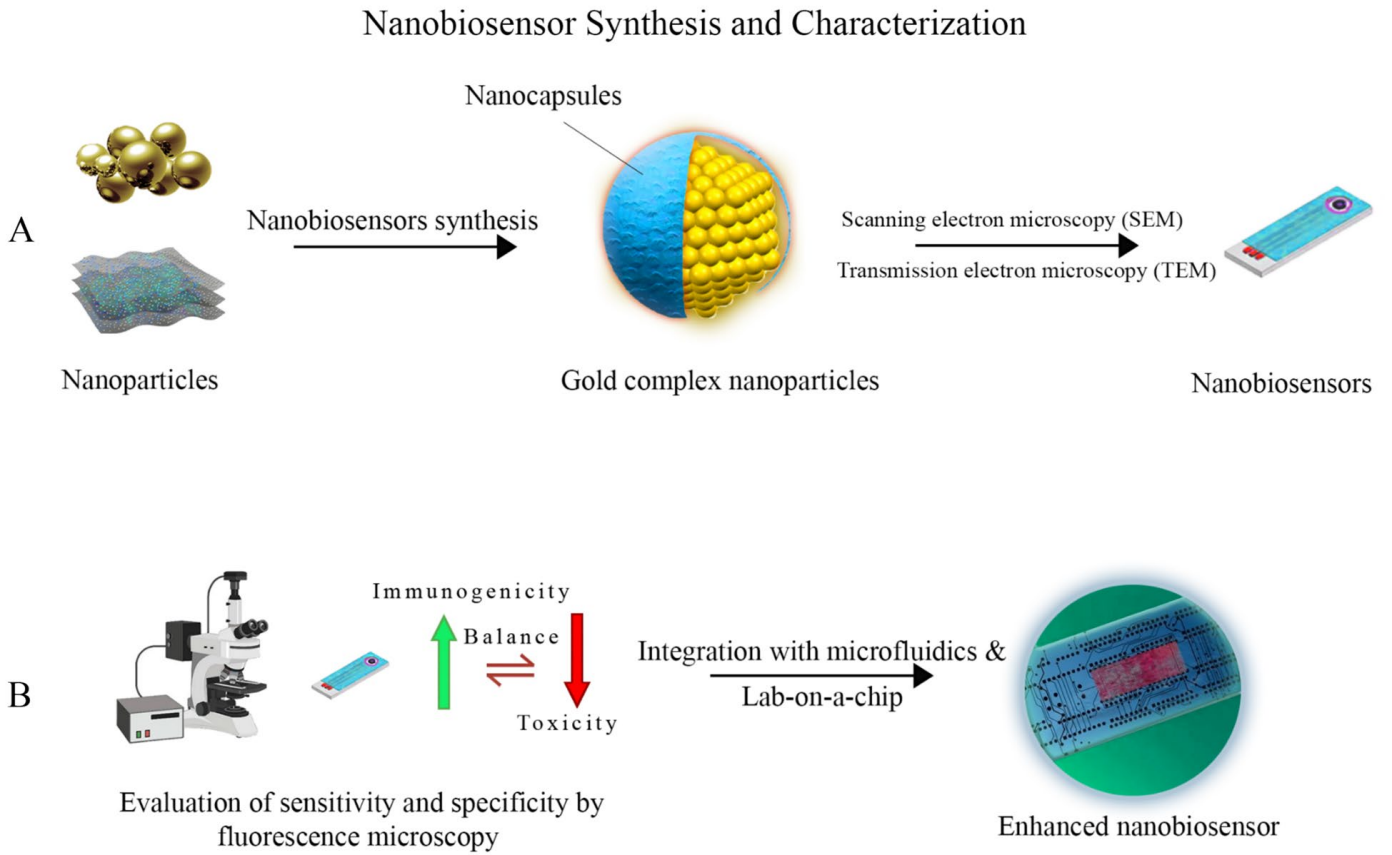
Overview of nanobiosensor fabrication and characterization. **A** Synthesis and characterization of nanobiosensors, highlighting essential techniques and processes involved. Functionalized with biological molecules such as antibodies or DNA strands for specific analyte detection. **B** Integration with microfluidics and lab-on-a-chip technologies enhances efficacy and allows quick and multiplexed analysis

The scanning electron microscopy (SEM) and transmission electron microscopy (TEM) methods have been used to characterize the size and morphology of the nanobiosensors [62]. The reliability and particle properties are evaluated utilizing zeta potential (ZP) measurements, and the sensitivity and specificity of the nanobiosensors are assessed using fluorescence microscopy and electrochemical impedance spectroscopy (EIS) [50, 63, 64]. The development of innovative nanobiosensors platforms with enhanced sensitivity, selectivity, and functionality for many medical diagnostic applications results from ongoing multidisciplinary collaboration among nanotechnology, biology, and engineering researchers [65] (Fig. 1).

Due to their sensitive, specific, and rapid detection capabilities, nanobiosensors will soon become one of the most significant breakthroughs in parasitic infection diagnosis and management [66–69]. Due to the incorporation of nanotechnology and biological recognition elements, nanobiosensors provide an accurate and early diagnosis of parasites at the molecular level, thus providing a powerful tool for early detection and treatment [42, 70]. However, multidisciplinary collaboration among nanotechnology, biology, and engineering researchers will drive innovation in nanobiosensors platforms for diagnosing zoonotic parasitic infections.

Nanobiosensors have been developed for a broad range of parasitic infections [71]. Each type of nanobiosensor utilizes different detection mechanisms with different types of nanomaterials and transduction principles to target specific parasites. In particular, electrochemical nanobiosensors can detect changes in electrical signals when attached to parasitic antigens or DNA based on the electrochemical properties of nanoparticles [72–74].

Interfaces between parasitic molecules and a specific probe are detected by nanobiosensors using the optical properties of nanoparticles, which are accomplished using several different methods, such as surface plasmon resonance (SPR) and fluorescence resonance energy transfer (FRET), among others [75, 76]. In particular, SPR technology is highly sensitive, allowing nanobiosensors to detect even small changes in the refractive index [77–79]. Hence, it makes nanobiosensors the perfect solution for detecting small concentrations of parasitic biomarkers that would otherwise go undetected by conventional techniques [80, 81]. The fluorescence nanobiosensors use fluorescent nanoparticles such as QDs that emit light when they bind with the targets, producing a visible signal that can be easily measured and quantified [82–85]. In addition, magnetic nanobiosensors can analyze complex specimen and detect antigens with great sensitivity and specificity using magnetic nanoparticles in their structure [86–88]. Detecting parasites with the help of nanobiosensors is particularly useful, since they can efficiently isolate target molecules from blood specimen precisely.

Parasitic diseases are among the major health problems around the world, infecting millions of people and killing thousands every year [89–91]. In addition to imposing heavy costs on health systems, these diseases impose social and economic effects in endemic regions [91–93]. Among the important parasitic diseases are malaria, leishmaniasis, CE, taeniasis, and schistosomiasis, and the COVID-19 pandemic significantly impacted them [94, 95]. Table 1 compares nanobiosensors with conventional diagnostic tools (ELISA, PCR, microscopy) for parasitic diseases (Table 1).

**Table 1:** Nanobiosensors with conventional diagnostic tools (ELISA, PCR, microscopy) for parasitic diseases, evaluating sensitivity, specificity, cost, time-to-result, throughput, ease of use, portability, specimen preparation, and applications. Nanobiosensors excel in sensitivity, speed, and portability but face challenges in cost and standardization, whereas conventional methods vary in scalability and technical demands

Parameter	Nanobiosensors	ELISA	PCR	Microscopy
Sensitivity	Extremely high (detects low analyte concentrations, *e.g.*, femtomolar levels)	Moderate to high (depends on antibody affinity)	Very high (detects DNA/RNA at low copies)	Low to moderate (depends on parasite load and technician skill)
Specificity	Extremely high (target-specific probes reduce cross-reactivity)	High (if antibodies are well-optimized)	Very high (primers target unique sequences)	Moderate (morphological overlap may cause misidentification)
Cost	High (nanomaterial synthesis and functionalization increase cost)	Low to moderate (reagents are standardized)	High (equipment and reagents are expensive)	Very low (minimal equipment needed)
Time-to-result	Rapid (minutes to hours, real-time detection possible)	Hours (4–6 h for standard protocols)	Hours to days (includes extraction, amplification, and analysis)	Minutes to hours (specimen preparation and manual examination)
Throughput	High (multiplexing capability for simultaneous detection)	Moderate (96-well plates allow batch processing)	Low to moderate (limited by thermocycler capacity)	Low (manual process limits scalability)
Ease of Use	Requires technical expertise for fabrication and operation	Standardized protocols (suitable for labs)	Requires trained personnel and equipment	Simple but skill-dependent (prone to human error)
Portability	High (can be integrated into point-of-care devices)	Low (lab-bound equipment)	Low (requires thermocyclers, electrophoresis)	Moderate (portable microscopes exist but lack sensitivity)
Specimen Preparation	Moderate (direct detection from complex matrices is possible)	Moderate (may require pre-processing)	Extensive (DNA/RNA extraction needed)	Moderate (staining/concentration steps)
Applications	Field-deployable, personalized medicine, dynamic monitoring	High-volume screening, research	Gold standard for molecular confirmation	Routine screening in endemic areas

## Protozoan parasites

Zoonotic protozoan parasites such as *Plasmodium* and *Leishmania* are important to public health and veterinary medicine, because they can be transmitted between humans and animals and cause severe diseases. Protozoan parasites threaten human health and have a significant economic impact on the livestock industry. Control of protozoan parasite infections requires public awareness, prevention, and interdisciplinary cooperation in the medical, veterinary, and environmental fields.

### *Plasmodium*

There are more than 150 species of *Plasmodium* that can be found in a wide range of vertebrates, five of which are considered true parasites of humans, because they utilize humans as a natural intermediate host for their life cycles: *P. falciparum*, *P. vivax*, *P. ovale*, *P. malariae*, and *P. knowlesi* some of which are considered zoonotic or have been reported in animals. Approximately 249 million malaria cases and 608,000 malaria-related deaths are expected to occur worldwide in 2022, with 85 countries affected by malaria [96]. Female *Anopheles* mosquitoes transmit *Plasmodium* parasites to people when they feed on blood to produce eggs. Drug resistance is a major challenge that needs to be addressed to effectively control malaria [97]. *Plasmodium* species have become increasingly resistant to common chemical drugs, such as chloroquine and artemisinin [98], leading to a decrease in the effectiveness of treatments and an increase in the death rate [99, 100]. Hence, it is imperative to rapidly diagnose malaria and administer antimalarial drugs as soon as possible to reduce the complications and risk of transmission. Traditional methods of diagnosis include blood tests and microscopy [101–103]. However, thanks to their high sensitivity and accuracy, nanobiosensors can play an important role in rapid and effective malaria diagnosis [104]. Nanobiosensors can quickly detect the presence of pathogens in blood specimen and provide the result in a short time [105]. Nanobiosensors based on gold and silicon nanoparticles are among the most widely used in this field.

According to a previous study, a new generation of electrochemical nanosensors using a gold electrode (Au) supported on metal oxide nanoparticles [106]. Based on the testing of the developed nanosensor, it was determined to detect malaria biomarkers. It also determined the optimum conditions under which maximum detection and quantification occurred. In addition, another study selected *P. falciparum* histidine-rich protein 2 (*Pf*HRP2) as a biomarker for malaria diagnosis and detection [107]. To determine whether the immunoreagent would be suitable for sensor development, an ELISA test was first developed. To develop the immunosensor for *Pf*HRP2, researchers first selected, characterized, and evaluated a gold-based sensor with a counter and an Ag/AgCl reference electrode. A monoclonal antibody specific for* Pf*HRP2 was immobilized with the sensor as a capture receptor. Assays were constructed using sandwich ELISA, with horseradish peroxidase (HRP) acting as the enzyme label and 3, 3′, 5, 5′tetramethylbenzidine dihydrochloride (TMB) and H_2_O_2_ acting as electrochemical signals. With the optimized and characterized assay and sensor, *Pf*HRP2 demonstrated a low limit of detection (LOD) in buffer samples and 100% spiked serum samples. Gold nanoparticles conjugated to detection antibodies–enzymes amplified the assay signal, and a detection limit of 36 pg/mL was achieved in buffer samples and 40 pg/mL in serum samples.

Several species-specific DNA nanosensors were designed to detect *P. falciparum*, *P. malariae*, and *P. ovale* using EIS, a label-free DNA-based nanosensor methodology. Even though the three species-specific genosensors’ detection limits might be lower than previously reported malaria genosensor detection limits, the LODs for the three species-specific genosensors were between 18.7aM and 43.6aM. In addition, quantitative real-time PCR (qPCR) assays using purified genomic DNA and paired whole blood lysates from clinical samples were applied to compare the diagnostic performance of the three genosensors qPCR assays. Interestingly, all three *Plasmodium* species detected by the genosensors were correctly identified by the qPCR-positive purified genomic DNA samples, indicating that genosensors are 100% able to identify each of the species of Plasmodium. Approximately 66.7–100.0% of the specificities of each genosensor were found, with a therapeutic turnaround time (TTAT) of less than 30 min, comparable to the TTAT of current PoC diagnostic tools for malaria used today [108].

Detecting an elevated level of heme in the blood serum is one of the easiest ways to detect a malaria infection quickly. The low cost, good biocompatibility, and biodegradability of albumins make them popular among bioengineers, because they are widely used in bioengineering. Glutaraldehyde cross-linked bovine serum albumin (BSA) exhibits strong autofluorescence due to its cross-linking with glutaraldehyde and is reported to form a suspension of fluorescent nanoparticles with an average diameter of 40nm, which are exceptionally fluorescent when compared with ordinary BSA [109]. In addition, an inexpensive and portable optogenetic biosensor was developed to detect and amplify malarial mitochondrial DNA rapidly [110]. Using nucleic acid scaffolds containing endonucleolytic DNAzymes and their substrates, bioresponsive magnetic nanoparticle assemblies are constructed in which magnetic nanoparticles can be released for optogenetic quantification when activated in the presence of target DNA. When exposed to target DNA, magnetic nanoparticles self-disintegrate. This process is particularly effective in causing padlock probe ligation by target molecules, which results in a homogeneous cascade reaction involving rolling circle amplification enhanced by nicking, nucleic acid recycling assisted by DNAzymes, and magnetic assembly disintegration driven by strand displacement.

Furthermore, a malaria biomarker, *Plasmodium* lactate dehydrogenase (pLDH), was found to interact with pL1 aptamers that were capable of inhibiting the production of *P. vivax* *Pv*LDH and *P. falciparum* *Pf*LDH. Using the aptasensor system, it was possible to detect low levels of pLDH proteins using this method [111].

Using magneto-enriched sample preparation, an uninstrumented lateral flow strip detection method based on stimuli-responsive nanoparticles was developed to detect model antigens from spiked pooled plasma. Gold-labeled biomarker half-sandwich can be purified and enhanced with the integrated reagent system for direct application to lateral flow test strips. This linear diblock copolymer includes a NIPAm-co–N,N-dimethylaminoethylacrylamide block bound to gold, which is a thermally responsive segment in the poly (N-isopropylacrylamide) (pNIPAm) segment. pNIPAm–AuNPs co-decorated with streptavidin were produced by functionalizing gold nanoparticles with a diblock copolymer and bioconjugation. Using pooled plasma samples spiked with pan-aldolase and *Pf*HRP2; these AuNPs efficiently complexed biotinylated capture antibody reagents. A 10nm thermally responsive magnetic nanoparticle decorated with pNIPAm was used to purify and enrich the gold-labeled biomarker half-sandwich. An applied thermal stimulus and a magnetic field generated large aggregates of iron oxide nanoparticles with pNIPAm-coated NP half-sandwiches that were magnetophoresed and separated efficiently from bulk serum [112].

Using a gold nanoparticle-enhanced platform (GNP), a recent study demonstrated the possibility of detecting *P. falciparum*-infected red blood cells without using any labels. On screen-printed electrodes, GNPs were electrodeposited to serve the dual purpose of immobilizing antibodies and enhancing electronic signals by forming a well-controlled matrix. As a result of their binding to the cell-reactive antibodies immobilized on the electrode, the infected red blood cells were identified by measuring the changes in electrical parameters that occurred owing to their binding to the antibodies. Approximately 10^2^ to 10^8^ red blood cells per mL were found to have good sensitivity to the electron transfer resistance, and a linear relationship between the logarithm of the number of infected cells and the electron transfer resistance [113].

**Fig. 2:**
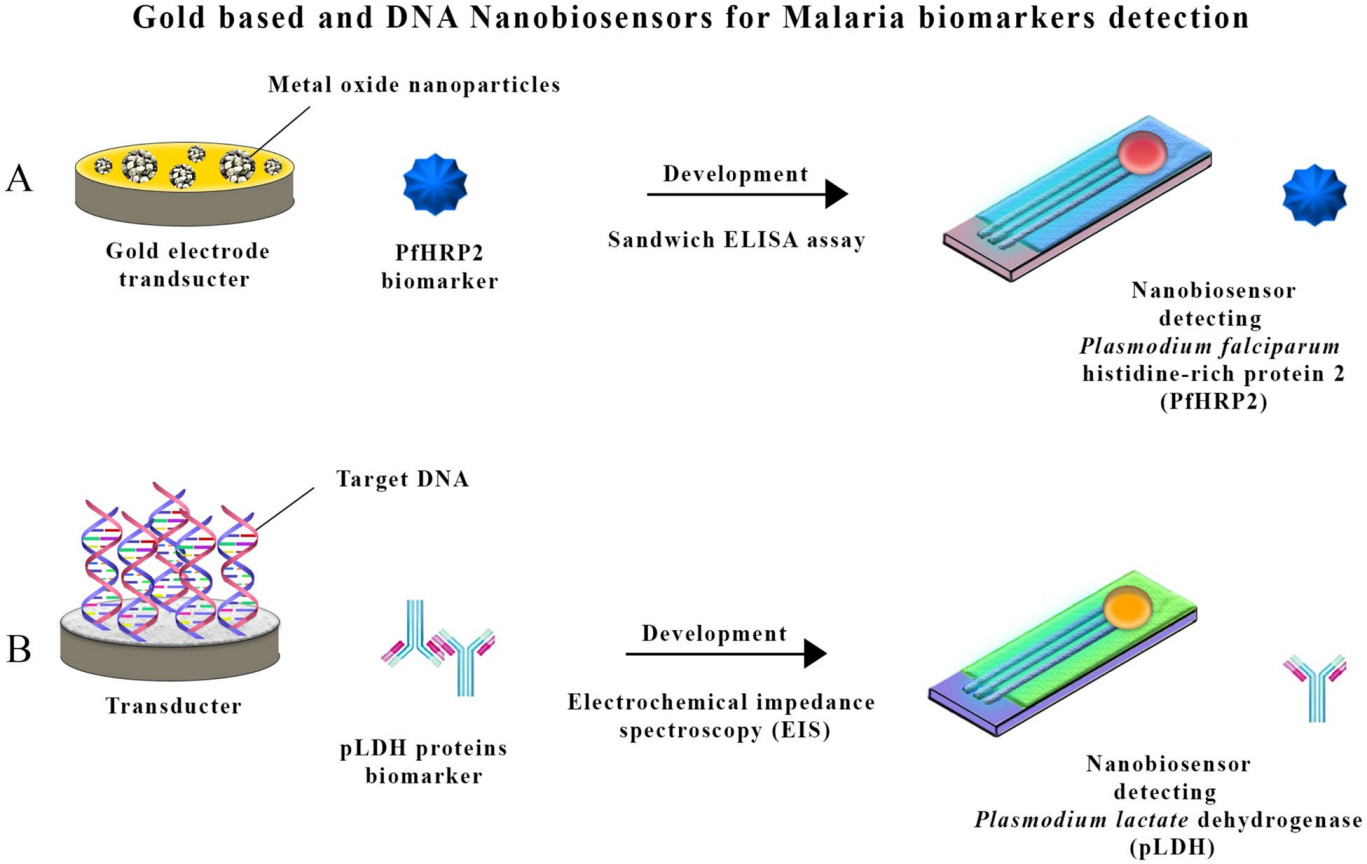
Overview of gold-based and DNA nanobiosensors for malaria detection. **A** This point shows an electrochemical nanobiosensor with a gold electrode supported on metal oxide nanoparticles, depicting the detection process of malaria biomarkers. Moreover, diagrams show the ELISA test setup targeting *P. falciparum* histidine-rich protein 2 (*Pf*HRP2) and the aptasensor system detecting *Plasmodium* lactate dehydrogenase (pLDH) proteins. This part features gold-based sensors, highlighting the immobilization of monoclonal antibodies and the sandwich ELISA setup. **B** This section shows species-specific DNA nanosensors for detecting *P. falciparum*, *P. malaria*, and *P. ovale*, emphasizing electrochemical impedance spectroscopy (EIS) in detection

Moreover, there was an attempt for a novel approach to introduce biomolecular recognition elements (BREs) into molecular recognition systems using a single-layer, two-dimensional nanomaterial (WSe2) [114]. It effectively detects schizonts with refractive index (RI) = 1.371, trophozoites with RI = 1.381, and ring parasites with RI = 1.396 at 633 nm with the optimized sensor design. The LRSPR biosensor shows an improvement of up to ten times detection accuracy (DA) over conventional surface plasmon resonance (CSPR). It also significantly improves the imaging figure of merit (IFOM) by 40 to 86.55 times and its imaging sensitivity for sequential detection of malaria stages. Schizonts, trophozoites, and ring stages can be detected using COMSOL multiphysics^®^ simulation software with penetration depths of 258.71 nm, 276.85 nm, and 373.04 nm. Another research developed in-house antibodies against the conserved C-terminal 105 amino acids of the *Pf*HRP-II biomarker using an electrochemical immunosensor technique [115]. This sensor utilizes highly redox-active thionine (Th) immobilized on a carbon nanofiber (CNF)-based chemically modified electrode (CME) platform. The immunosensor showed remarkable sensing signals ranging from 250 pg/mL to 100 ng/mL *Pf*HRP-II, with a high current sensitivity of 0.813 μA/ng mL^–1^ (Table 2) (Fig. 2).

**Table 2:** Current status of nanobiosensors development on the diagnosis of malaria

Methodology	Biomarker	Results	Reference
Electrochemical nanosensors using a gold electrode supported on metal oxide nanoparticles	*Pf*HRP2	Maximum detection and quantification occurred	[106]
A gold-based sensor with a counter and an Ag/AgCl reference electrode for *Pf*HRP2 as a biomarker	*Pf*HRP2	A detection limit of 36 pg/mL and 40 pg/mL was achieved in buffer and serum samples	[107]
Glutaraldehyde cross-linked BSA	heme	Form a suspension of fluorescent nanoparticles with an average diameter of 40 nm	[109]
Portable optogenetic biosensor using nucleic acid scaffolds containing endonucleolytic DNAzymes	DNA	Results in a homogeneous cascade reaction involving rolling circle amplification enhanced by nicking, nucleic acid recycling assisted by DNAzymes	[110]
pL1 aptamers	pLDH	Detect low levels of pLDH proteins	[111]
Using a GNP without using any labels	*P. falciparum*-infected red blood cells	10^2^ to 10^8^ red blood cells per mL were found	[113]
BREs into molecular recognition systems using WSe_2_	Schizonts, trophozoites, and ring stages	Detecting schizonts with refractive index (RI) = 1.371, trophozoites with RI = 1.381, and ring parasites with RI = 1.396 at 633nm	[114]
Electrochemical immunosensor technique utilizing highly redox-active thionine (Th) immobilized on a carbon nanofiber (CNF)-based	*Pf*HRP-II	A remarkable sensing signals ranging from 250 pg/mL to 100 ng/mL *Pf*HRP-II, with a sensitivity of 0.813 μA/ng mL^–1^	[115]

Among the reported methods for detecting *Plasmodium* species, electrochemical immunosensors based on antigen–antibody interactions while demonstrating acceptable sensitivity, often involve complex multistep protocols and labeling agents, which can limit their applicability in rapid or PoC settings. In contrast, label-free impedance-based approaches, which do not rely on molecular markers, have achieved highly sensitive detection across multiple *Plasmodium* species within a short assay time and have shown complete agreement with qPCR results, highlighting their potential for clinical diagnostics. Efforts to simplify detection through devices with magnetic particle-assisted target enrichment further emphasize the move toward more field-deployable formats. However, such systems may still face challenges with analytical accuracy or preprocessing requirements. Optical methods utilizing SPR have demonstrated superior resolution in differentiating intraerythrocytic stages of the parasite. However, their dependence on advanced optical instrumentation restricts their use outside laboratory environments. Label-free impedance methods offer a more favorable balance between sensitivity, specificity, speed, and operational simplicity, whereas other technologies present complementary advantages depending on the diagnostic context.

### *Leishmania*

Leishmaniasis is another parasitic disease transmitted by *Leishmania* parasites through the bite of an infected female phlebotomine sand fly [116–118]. Leishmaniasis can appear in cutaneous leishmaniasis (CL), visceral leishmaniasis (VL), and mucocutaneous forms, with an estimated 700,000 to 1 million new cases annually, mostly in tropical and subtropical regions [119]. Drug resistance in leishmaniasis is also one of the main challenges worldwide in treating and controlling leishmaniasis [120]. Many *Leishmania* spp. have become resistant to common chemical drugs such as pentavalent antimonials, leading to decreased therapeutic effects and subsequently increased disease cases [121–125]. Rapid diagnosis of leishmaniasis is very important to prevent serious complications and even mortality [126]. Traditional methods of diagnosis include blood tests and tissue sampling, which require much time and can also be challenging, and have low accuracy [127, 128]. A timely diagnosis must be made to manage and treat the disease effectively [129]. If leishmaniasis is not diagnosed promptly, severe complications, including disfiguring skin lesions, organ damage, and even death, might occur, particularly in VL [130, 131]. When leishmaniasis is detected early, it can also be controlled, and vector control measures can be implemented to reduce transmission rates and spread [132]. Nanobiosensors can aid in the quick and accurate detection of parasites in clinical specimen [32, 133], playing an essential role in disease management [71]. Nanobiosensors based on carbon nanotubes and magnetic nanoparticles are among the effective tools in this field.

Using colorimetric and amplification methods aimed at parasitic internal transcribed spacer 2 (ITS2) fragments, AuNP-Probe conjugates to detect *Leishmania* spp. were designed. First, 10 µL of DNA was hybridized with 4µL of probe and 5µL of 0.2N HCl added (non-amplification method). After the first method, 5µL of AuNP, 5µL of 0.2N HCl, and thiolated primers were used to amplify the DNA by PCR. *Leishmania major*, *L. tropica*, and *L. infantum* were detected by amplification and non-amplification methods at 32 fg/µL and 16 fg/µL. Visceral leishmaniasis detection sensitivity was 96% in the non-amplification method, and 100% in the amplification method, and for cutaneous leishmaniasis (CL) was 98% and 100%, respectively. The results showed that the sensitivity of the amplification method was the same as that of RT–qPCR, while that of the non-amplification method was lower [133].

Furthermore, a powerful method was developed to detect *L. major* kinetoplast DNA (kDNA) using small gold nanoparticles in conjunction with non-protein-coding DNA probes from the minicircle kinetoplast segments. The test was determined to diagnose *L. major* from various non-*Leishmania* species with a detection limit of 7.0 pg/μL. Genomic DNA extracted from clinical samples and *L. major* was identified using the PCR-free assay [134].

Researchers have simplified the detection of PCR products by combining nucleic acid lateral flow with functionalized gold nanoparticles. The kDNA of *Leishmania* species was amplified from canine blood samples using an amplification reaction. This resulted in the occurrence of a red test zone. As a result of the visual detection, the process took 20 min to complete. A significant amount of optimization led to the detection of 100 fmol of target DNA after extensive optimization [135].

To detect the DNA of *L. infantum* in dog blood samples, an improved triple-line lateral-flow assay (LFA) was designed and successfully used to detect its DNA. It was discovered that a more efficient method of identifying anti-FITC antibodies is to use AuNPs conjugated to polyclonal secondary antibodies. Because the secondary antibodies possess a polyclonal nature, multiple bindings are possible to the primary antibodies, significantly enhancing the AuNP plasmonic signal. In addition, a biochemical method of avoiding false negatives was developed that controlled the endogenous control of the amplified dog 18S rRNA gene. The results showed that 0.038 spiked *Leishmania* parasites were detected per DNA amplification reaction [136].

With an array of chemically sensitive gas sensors, a simple-to-use, noninvasive method of diagnosing CL in humans by taking measurements of volatile organic compounds in exhaled breath was designed. Interestingly, one of the sensors had 100% accuracy, 100% sensitivity, and 100% specificity for detecting human CL based on CuNPs functionalized with 2-mercaptobenzoxazole [137].

The development of DNA biosensors using nickel oxide (NiO) films synthesized by the sol–gel method was demonstrated for the diagnosis of VL using NiO films synthesized by the sol–gel method. Using 18S rRNA gene sequences from *L. donovani*, 23mer DNA sequences (oligonucleotides) were used to develop a *Leishmania*-specific sensor. X-ray diffraction and scanning electron microscopy measurements have demonstrated the formation of nano-structured NiO. Furthermore, *Leishmania* DNA was immobilized through single-strand UV–visible, Fourier transform infrared spectroscopy (FTIR), and SEM. In the presence of methylene blue redox dye, differential pulsed voltammetry is used to study the response of a small subunit DNA/NiO/ITO bioelectrode. A linear response was observed over a wide range of concentrations, within a 10% variation in complementary target genomic DNA concentration [138].

Using an ultrasensitive electrochemical DNA biosensor, researchers identified unlabeled *Leishmania* parasites without the employment of PCR, using an electrodeposition method to deposit gold nano leaves and spermidine to act as a shape-directing agent. During biosensor production, gold nano leaves were immobilized with a DNA probe specifically designed for *L. major*, and methylene blue was used as a marker substance. It was then found that the complementary single-stranded sequence hybridized with the biosensor under selected conditions. There was a high level of selectivity among the biosensors when it could distinguish *L. major* from a non-complementary sequence oligonucleotide and *L. tropica* [139].

It has been demonstrated that a recombinant antigen, rLci2B, immobilized on quartz crystal electrodes, is capable of highly sensitive piezoelectric immunosensors for anti-*Leishmania* antibodies. Due to the increase in surface area and bond stability, a Nafion film was utilized to build up the electrode surface. AuNPs were used to recover gold from the film to promote a significant amount of rLci2B. Higher sensitivity and reproducibility were obtained with the AuNP-based immunosensor than with its cysteamine-based counterpart, using a cysteamine-based immunosensor without AuNP [140].

Moreover, a thiolated carbon nanotube (ThNT) transducer was developed based on electrical impedance spectroscopy to detect femtomolar DNA levels from *Leishmania* species. Single-stranded DNA probes functionalized with carboxyl groups were covalently attached to ultrathin Au films anchored to carboxyl-functionalized ThNTs. As a result of forcefully disengaging the ThNTs during their covalent immobilization with an external magnetic field, the genosensors exhibited less resistance than when immobilized naturally. It was found that the sensors could linearly detect parasite DNA between 0.1 and 98.3 fg/μL under disentangled conditions. Based on canine genomic samples extracted directly from the blood of infected animals, the lower limit corresponds to a CL DNA concentration of 15 fM [141].

In addition, cadmium selenite quantum dot probes were combined with magnetic beads to detect *Leishmania*-specific surface antigens and DNA. Magnetic bead capture probes were used to isolate the targeted molecules from the solution. In contrast, quantum dot detection probes were used to confirm the presence of the targeted molecules in the solution. Based on the results of an assessment of 55 cultured isolates of various microbial pathogens, it has been determined that the sensitivity and specificity of this method are 100%. For the DNA and protein of *Leishmania* parasites, the low detection limit was determined at 3125 ng/mL and 10^3^ cells/mL [142].

Various peptides were investigated for their ability to detect antibodies against *Leishmania* in human serum and dog specimen [143]. Graphene oxide (GO) and graphene quantum dots (GQD) are graphene compounds with different solid-binding domains. While both peptides have the same recognition site, they differ in their ability to bind to the graphene oxide and graphene quantum dots spontaneously. The cyclic voltammetry and differential pulse voltammetry techniques were employed to better understand the electrochemical behavior of each stage of the assembly procedure. Its relationship with the solid-binding domain and the anchoring material was evaluated to determine their functions during the assembly procedure. The graphene affinity peptide (395-G) demonstrated increased reproducibility and selectivity when coupled with the graphene affinity peptide. A cutoff value of 82.5% at a 95% confidence level was used to distinguish between adverse and positive responses to human serum specimen under experimental conditions.

**Fig. 3:**
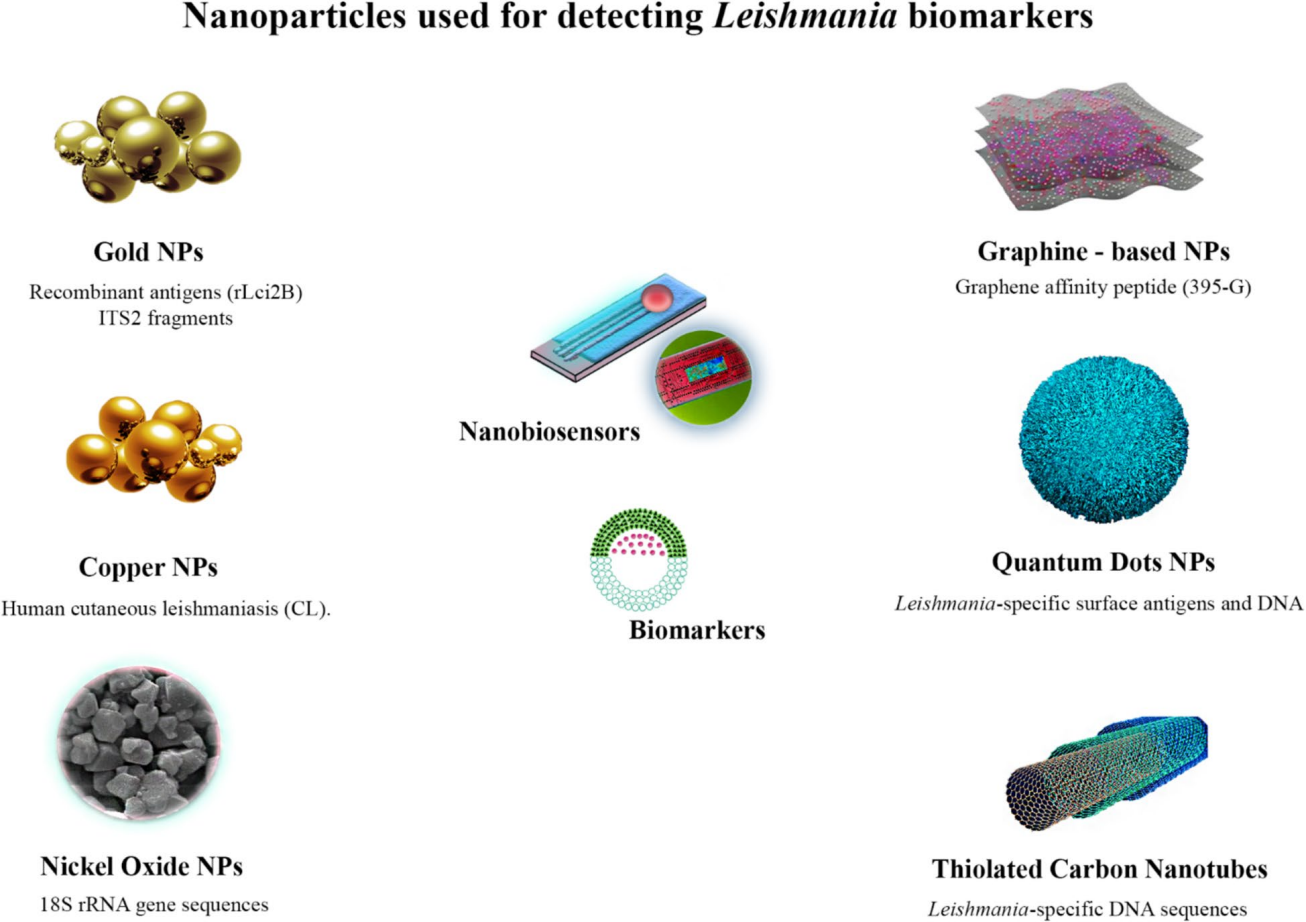
Current illustration depicts the use of various nanoparticles to detect biomarkers associated with *Leishmania* parasites. Gold nanoparticles are used with non-protein-coding DNA probes to detect *L. major* kDNA. Cadmium selenite quantum dots probes are combined with magnetic beads to detect *Leishmania*-specific surface antigens and DNA. Copper nanoparticles functionalized with 2-mercaptobenzoxazole detect CL based on volatile organic compounds in exhaled breath. These NPs offer sensitive and specific detection methods for various forms of leishmaniasis, aiding in timely diagnosis and effective disease management

Another study developed an immunosensor based on a CoFe_2_O_4_–C_60_ nanocomposite decorated with a sensitive A2 peptide antigen to detect anti-A2 antibodies for diagnosing VL [144]. The immunosensor demonstrated a linear range of 10^−10^–10^−1^ µg/mL, with a detection limit of 30.34 fg/Ml (Table 3) (Fig. 3).

**Table 3:** Current status of nanobiosensors development on the diagnosis of leishmaniasis

Methodology	Biomarker	Results	References
Colorimetric AuNP-Probe	ITS2 fragments	Detection limit of 32 fg/µL and 16 fg/µL	[133]
AuNPs with non-protein-coding DNA probes	kDNA	Detection limit of 7.0 pg/μL	[134]
Combining nucleic acid lateral flow with AuNPs	kDNA	Detection of 100 fmol of target DNA	[135]
Triple-line LFA AuNPs conjugated	DNA and 18S rRNA	0.038 spiked *Leishmania* detected per DNA amplification reaction	[136]
Chemical sensors based on ligand-capped CuNPs for diagnosing CL	Measurements of volatile organic compounds in exhaled breath	100% accuracy, 100% sensitivity, and 100% specificity for detecting human CL	[137]
DNA biosensors using NiO films synthesized by the sol–gel	18S rRNA	A linear response is observed over a range of 2 pg/ml to 2 μg/ml concentrations within a 10% variation in complementary target genomic DNA concentration	[138]
Ultrasensitive electrochemical DNA biosensor based on gold nanoleaves	DNA	The biosensor could detect synthetic DNA targets ranging from 1.0 × 10^−10^ to 1.0 × 10^−19^ mol/L with an LOD of 1.8 × 10^−20^ mol/L and genomic DNA in a range of 0.5–20 ng/μL with an LOD of 0.07 ng/μL	[139]
AuNPs-based immunosensor	rLci2B	Higher sensitivity and reproducibility were obtained	[140]
ThNT transducer to detect femtomolar DNA levels	DNA	Detecting parasite DNA between 0.1 and 98.3 fg/μL	[141]
Cadmium selenite quantum dots combined with magnetic beads	*Leishmania*-specific surface antigens and DNA	For DNA and protein, the low detection limit was 3125 ng/mL and 10^3 ^cells/mL	[142]
An immunosensor based on a CoFe_2_O_4_–C_60_ nanocomposite decorated with a sensitive A2 peptide antigen	Anti-A2 antibodies	The immunosensor demonstrated a linear range of 10^−10^–10^−1^ µg/mL, with a detection limit of 30.34 fg/Ml	[144]

The main strengths of AuNP and LFA-based methods are their simplicity of implementation, speed of response (20–60 min), and usability in field environments without the need for complex infrastructure. The sensitivity of the three-line LFA with AuNP conjugated to polymorphic antibodies (LOD < 0.04 parasites after PCR) and the AuNP–Probe system for the ITS2 sequence (LOD 32 fg/µL without amplification) is very suitable for field detection; however, the dependence of these methods on pre-amplification steps in samples with low parasite load and the possibility of error in colorimetric reading are their weaknesses. AuNP colorimetric assays reduce errors due to interference from clinical matrices without the need for sophisticated equipment. However, they do not achieve optimal sensitivity and reproducibility at high levels compared to electrochemical techniques and EIS-based transducers.

In contrast, electrochemical biosensors, including NiO films with a 23mer 18S rRNA probe (linear range 2pg/mL–2µg/mL) and a ThNT–Au transducer with EIS (LOD 0.1fg/µL), offer the highest sensitivity and reproducibility and are ideal for precise analyses in research laboratories. Although the need for specialized equipment and complex sample processing (DNA extraction, control of electrochemical conditions) are obstacles to their field application, the ability to detect at the molecular level and an error of ≤ 10% over a wide range of concentrations make this group recommended for quantitative studies. Furthermore, nanomagnetic–QD covalent systems and CoFe₂O₄–C₆₀ nanocomposite systems with the ability to simultaneously separate and detect multiple targets (DNA, antibodies) in a single platform, with low LOD and multiplex potential, although they require complex fabrication and careful washing steps, provide significant prospects for the development of multiplex techniques.

## Helminthiasis

Millions of people all around the world are affected by helminthic infections, which are caused by parasitic worms, such as nematodes, cestodes, and trematodes [145–148]. It is essential to ensure that a prompt diagnosis is made for helminthic infections to initiate the treatment rapidly to reduce the risk of anemia, delayed growth, and cognitive development, especially in children [149, 150]. The following sections will discuss using nanobiosensors to diagnose helminthic infections.

### Cystic echinococcosis

Cystic echinococcosis is a serious health problem resulting from *E. granulosus* sensu lato cestode infection. As a result of the ingestion of parasites’ eggs in contaminated food or water humans become infected with CE [151]. Cystic echinococcosis leads to the formation of cysts in various organs, particularly in the liver and sometimes in the lungs of patients [152, 153]. Treatment of CE uses less invasive approaches, such as chemical antiparasitic drugs, the most commonly used of which is albendazole, or more invasive approaches like surgery [154, 155]. Rapid and early diagnosis of CE is critical to prevent serious complications and effective treatment. Diagnostic methods include medical imaging, such as ultrasound and computed tomography (CT), which require advanced equipment [156–159]. Nanobiosensors can help to diagnose CE quickly and accurately [160].

In previous research, an improved immuno-dot blot assay was presented to diagnose CE. The approach involved forming a sandwich complex using protein A between a gold nanoprobe and hydatid cyst antigen B (AgB). Protein A was conjugated to chitosan–gold nanoparticles. At the same time, AgB is immobilized on a nitrocellulose membrane. Sera samples and gold nanoprobes were added, and the naked eye quickly detected positive signals. Signal intensity correlated with the concentration of anti-*Echinococcus granulosus* antibodies, antibody titer in sera samples, and AgB concentration on the membrane. The minimum concentrations for protein A conjugation and Ag B coating were 0.5 and 0.3 mg/mL [161].

### Taeniasis/cysticercosis

Taeniasis is a gastrointestinal infection caused by three species of tapeworm. The most common species of tapeworm are *T. solium* (pork tapeworm), *T. saginata* (beef tapeworm), which are found throughout the world, and *T. asiatica*, which is located mainly in China, Taiwan, Indonesia, and Thailand, but is also found in Asia [162–164]. Most people get taeniasis by consuming undercooked meats, infected beef, pork, or viscera of pigs, which have been contaminated with the parasite's larvae (cysticerci). When cysticerci form in various organs of the body (cysticercosis), including the central nervous system (CNS), neurological symptoms (neurocysticercosis) can develop, such as epileptic seizures [165]. Rapid diagnosis of taeniasis is crucial to prevent serious complications and effective treatment [166]. Traditional methods of diagnosis include fecal examination and medical imaging, which require time and equipment [167].

A rapid and targeted biosensor was designed to identify *T. solium*, a parasite accountable for the pathogenic condition known as neurocysticercosis, which impacts the CNS [76]. An AuNP dispersed in a colloidal suspension is used to source the biosensor's localized surface plasmon resonance (LSPR) technology. Using these AuNPs, the immuno-capture effect was achieved through modification and antibody activation. A Turkevich and seed-mediated growth technique was used to produce the AuNPs. Multiple concentrations of *T. solium* antigen were tested to determine the detection and dose–response profile. The LOD of the antigen was less than 0.1 µg/mL based on the low antigen concentrations.

### Schistosomiasis

Schistosomiasis is a chronic disease throughout the world, and the infection occurs when freshwater snails release larvae of the parasite, which penetrate the skin of their final host. There have been reported cases of schistosomiasis transmission in 78 countries. Schistosomiasis is traditionally diagnosed by fecal or urine specimen analysis, which can be used to identify parasite eggs. As part of the diagnostic process, antibodies and/or antigens found in blood or urine specimen can also be used to diagnose schistosomiasis [168]. Nanobiosensors can help detect schistosomiasis patients quickly and accurately [169].

**Fig. 4:**
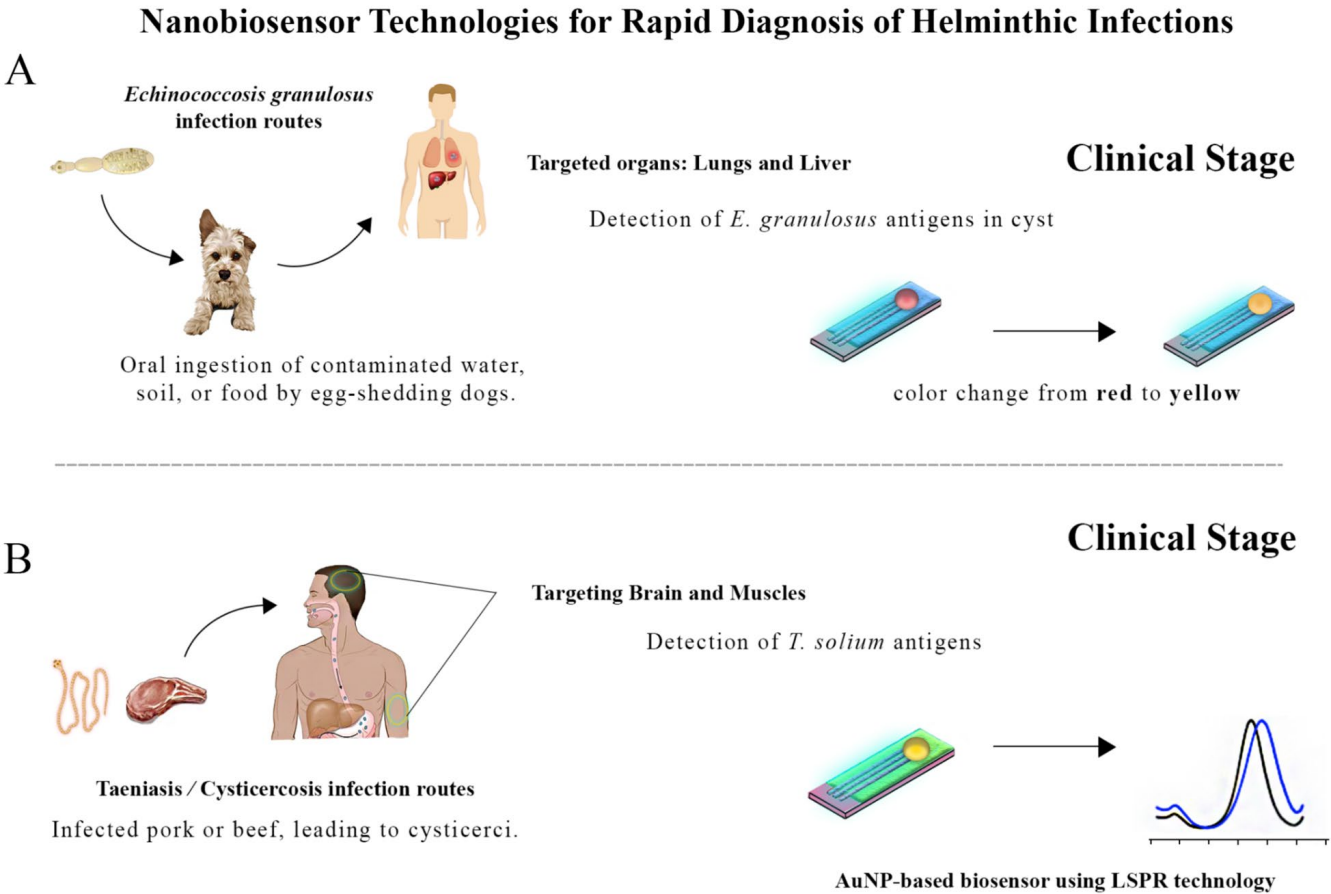
Application of a nanobiosensor for the rapid diagnosis of helminthic infections. Panel A highlights the infection routes of *Echinococcus granulosus*, targeting the lungs and liver, with detection focused on antigens within cysts. The clinical stage is indicated by a color change from red to yellow. Panel B details the infection routes of taeniasis/cysticercosis, transmitted through infected pork or beef, leading to cysticerci formation. The nanobiosensor targets the brain and muscles, detecting *T. solium* antigens, and employs AuNP-based biosensors utilizing localized surface plasmon resonance (LSPR) technology for diagnosis

A study developed a new screen-printed immunosensor to detect *S. mansoni* ABs [170]. The nanocarbon working surface of a screen-printed electrode was coated with soluble worm antigens (SWA) using glutaraldehyde–chitosan cross-linkers. Cyclic and differential pulse voltammetry were used to evaluate the binding capacity of *S. mansoni* antibodies to the antigen-loaded screen-printed electrode. A repeatable linear relationship was seen in the calibration curve for binding *S. mansoni* ABs to the SWA-loaded screen-printed electrode, with concentrations ranging from 0.038 to 20 ng/ml. The acquired quantifiable response at nano-level concentrations of ABs indicates the potential use of this technology to design disposable screen-printed electrodes for diagnosing schistosome infections. Furthermore, an electrochemical analysis to detect the presence of *S. mansoni* DNA was established [171]. This method was achieved by utilizing a self-assembled monolayer of mercaptobenzoic acid (MBA), immobilizing nanostructures of AuNPs and magnetite nanoparticles (Fe_3_O_4__NPs). The hybridization procedure was observed using cyclic voltammetry (CV) and EIS. The biosystem under consideration can identify distinct nucleotide sequences of *S. mansoni* in serum samples and cerebrospinal fluid, even when the concentration of genomic DNA varies. The extended platform demonstrated a DNA detection limit of 0.685 and 0.781 pg/μL for cerebrospinal fluid and serum (Table 4) (Fig. 4).

**Table 4:** Current status of using nanobiosensors for helminths infection diagnosis

Parasite	Methodology	Biomarker	Results	References
*Echinococcus granulosus*	Immuno-dot-blot assay	Hydatid cyst antigen	Minimum concentrations: 0.5 mg/mL (protein A), 0.3 mg/mL (Ag B)	[161]
*Taenia solium*	LSPR biosensor using AuNPs	*T. solium* antigen	LOD: < 0.1 µg/mL	[76]
*Schistosoma mansoni*	Screen-printed immunosensor	*S. mansoni* antibodies	Calibration curve: 0.038–20 ng/ml	[170]
*Schistosoma mansoni*	Electrochemical analysis	*S. mansoni* DNA	LOD: 0.685 pg/μL (cerebrospinal fluid), 0.781 pg/μL (serum)	[171]

## Challenges

Nowadays, nanotechnology and nanobiosensors are used in various medical fields [172]. Nanobiosensors are recently used to detect parasitic infections and improved the detection accuracy and nanobiosensors had many advantages over traditional methods, such as ELISA and PCR. One of the most important advantages of nanobiosensors is their high sensitivity in detecting biological specimens [173, 174]. This is significantly improving conventional methods. For example, ELISA and PCR usually require specimens with specific antigen or parasite DNA concentrations for accurate detection. At the same time, nanobiosensors can provide more accurate results at lower concentrations and with a smaller specimen volume. In addition, nanobiosensors can often provide results in shorter times [175]. This is especially helpful in emergency and low-resource areas with limited diagnostic facilities. In terms of accuracy or specificity, although traditional methods such as PCR are generally recognized as the gold standard and have a high ability to specifically detect parasitic genes, nanobiosensors using smart materials and nanoscale engineering can reach a level of specificity that competes with molecular techniques. Although nanobiosensors have many advantages, they are also subject to challenges, such as higher research and development costs and the need for standardization [176, 177]. Since nanobiosensors are highly sensitive and specific in detecting infectious diseases, their mass production is scientifically and technically possible, but there are several economic and infrastructure challenges to overcome. Nanobiosensors require nanostructured materials and advanced technologies, thus complicating and increasing the cost of production compared to traditional diagnostic methods, such as ELISA or PCR [178, 179].

The advancements in nanotechnology, using smart materials, and nanomaterial engineering can enabled the optimization of production processes to reduce costs. Nanoparticle production methods such as chemical synthesis and 3D printing can reduce production costs and simplify mass production. Nanobiosensors have a significant advantage in terms of scalability, because they are portable, which is important for endemic and low-resource regions [180]. The importance of this feature in areas without advanced laboratory infrastructure cannot be overstated. Nevertheless, nanobiosensors still require extensive development and research costs to be designed and produced. As the development process progresses, advanced technologies and special equipment become available, which can increase the cost of production [181]. Furthermore, standardization and quality control in the production process are essential for ensuring the quality and accuracy of the detection of nanobiosensors [182, 183]. However, nanobiosensors production will decrease as production volume increases. This is due to the growing demand for rapid and accurate diagnosis in endemic regions and nanotechnology investments [184]. By collaborating with public and private organizations and international health organizations, it should be possible to mass-produce nanobiosensors on a large scale [185]. Although nanobiosensors are expensive initially, their advantages, such as high reliability, fast response times, and ease of transport, can potentially reduce total costs in the long term [186–188], helping them to become an outstanding choice for diagnosing parasites in endemic regions.

Nanobiosensors are suitable for PoC tests in remote areas, because they provide fast and accurate results [189, 190]. One of the main issues raised is these devices' portability and ease of use for non-expert users. Nanobiosensors can be made in small sizes and simple designs, making them suitable for use in remote areas without access to advanced infrastructure. Various nanobiosensors can run on batteries and are not required to be connected to complex laboratory systems to operate. Thus, this technology has the advantage of allowing testing to be performed on-site in a very short amount of time [191–193]. Even with the promise of achieving this goal, one of the key challenges is to ensure the device maintains its stability and diagnostic accuracy under multiple environmental conditions and conditions varying from one place to another. Among these variables are temperature changes, humidity changes, and the storage of nanomaterials that may have an adverse impact on the performance of nanobiosensors [194]. It is important to design devices that are easy to operate for non-experts, such as local health workers, and that can be used efficiently by them. One of the critical concerns is user-friendliness. However, one of the main challenges in developing these devices will be providing them with the ability to be both technologically sophisticated and easy to use for people unfamiliar with advanced technologies. In general, some nanobiosensors require basic data processing and specialized training [195]. However, innovations such as using smartphones as diagnostic platforms have helped solve this challenge.

In biological specimens, such as blood or feces, nanobiosensors can detect parasites’ biomarkers [43, 169]. However, some challenges, such as the presence of interfering substances and the complexity of compounds of these specimens, can limit the accuracy and precision of the diagnosis. Proteins, cells, lipids, and other large molecules in blood and feces can interfere with nanobiosensors and cause false positives or negatives. Consequently, nanobiosensors must undergo special testing to minimize interferences and guarantee diagnostic accuracy. There are several ways to overcome this challenge, including using highly selectable nanostructured materials. Specifically, these substances can bind to biomarkers found in parasites and ignore any molecules that will interfere with their binding. There are various methods for recognizing biomarkers on nanoparticle surfaces. One of these methods is using aptamers or specialized antibodies on nanoparticle surfaces as recognition layers [196–198]. Using these biomolecular films in complex specimens, parasite antigens or target molecules can be selectively bound to them without interfering with other compounds in the specimen. Furthermore, some nanobiosensors can also improve signal and reduce noise due to new techniques [199]. These techniques include applying gold nanoparticles or magnetic nanoparticles to amplify the signals [200]. Furthermore, these particles not only improve the detection sensitivity, but as they absorb and remove interference compounds, they can also help to improve the detection accuracy of complex specimen by increasing the detection sensitivity. In addition, some techniques are used to filter and separate biological specimen. Certain pre-processings are performed to remove more giant cells or nonspecific proteins, which means the sensor can only access the biomarkers of interest. Multisensor systems are also an excellent way to implement nanobiosensors capable of simultaneously detecting various biomarkers. This method has an advantage, because it reduces diagnostic errors and facilitates parasite detection even if interfering substances are present in the specimen. In some cases, nanobiosensors have been designed to simultaneously detect several stages of a parasite's life cycle. This results in a higher diagnostic accuracy even when specimen contain many different parasites simultaneously.

Management of nanomaterial toxicity, such as QDs or Au nanoparticles, is an important challenge [201–203]. Various approaches are being used to address these challenges by reducing or eliminating the toxic effects of these nanomaterials and improving the biocompatibility of these materials [204–206]. Modifying the surface of nanomaterials with biocompatible coatings is the simplest and most effective method. Using these coatings prevents nanoparticles from coming into contact with cells and biological tissues directly, and their toxicity is reduced. In this regard, biocompatible coatings may be used to isolate nanoparticles from the environment to minimize the unintended uptake of nanoparticles and their interaction with cells. Some biocompatible coatings, such as polyethylene glycol (PEG) or natural proteins, may also produce coatings [207]. In addition, these coatings are also capable of controlling the residence time of nanoparticles inside the body, thereby allowing the nanoparticles to leave the biological system at a much faster rate than before they were introduced. Nanomaterials’ size, shape, and composition are also important considerations when determining their toxicity [208–210]. A nanomaterial with a very small particle size can easily enter cells and interact with sensitive parts of the cells, such as the nucleus or mitochondria. In this manner, the cells can suffer damage as a consequence, resulting in their death. There is, therefore, an important approach to reducing nanotoxic effects that involves controlling the size and shape of nanoparticles so as to decrease their ability to penetrate into cells. To do this, it is necessary to achieve precise control of the size and shape of nanoparticles [211, 212]. For example, gold nanoparticles with moderate sizes and round and smooth shapes are usually less toxic, because they interact less with cellular structures. Non-toxic or low-toxicity nanoparticles are another key approach. Nanomaterials are inherently toxic, so efforts are being made to replace them with less hazardous nanoparticles [213]. For example, instead of cadmium-based quantum dots, which are toxic, silicon- or carbon-based quantum dots can be used, which are less toxic and can still provide the optical and electrochemical properties required for biodiagnostics.

One of the features of nanobiosensors that makes them attractive is their potential for multiplexing, especially for the simultaneous detection of multiple parasitic infections. The multiplexing technique involves simultaneously detecting several biomarkers or pathogens in a single specimen, which is useful when diagnosing parasitic diseases that often coexist in endemic regions. Since nanobiosensors can be customized to meet specific diagnostic needs and are highly sensitive, they are believed to be the ideal platform for this type of multiple diagnostics. With nanobiosensors, various biomarkers can be detected simultaneously by binding at various levels of functionality and using different nanoparticles. For instance, a gold nanoparticle or quantum dot can be designed and engineered separately based on the biomarkers associated with different parasites, such as *Plasmodium* spp., *Leishmania* spp., or *Schistosoma* spp. A system like this would be able to detect several parasites simultaneously by attaching each nanoparticle to a specific antibody or aptamer that corresponds to the corresponding biomarker. This would make it possible to detect several parasites simultaneously. Furthermore, in addition to their unique optical and electrochemical properties, nanoparticles also help in the simultaneous detection of multiple targets in a single sensor by utilizing capabilities such as detecting different signals based on wavelengths or electrical spectra. While nanobiosensors are a promising technology for multiplex detection, they still have some technical challenges. A significant challenge in biomarkers is preventing signal interference between them, which can be very challenging. The different optical or electrical signals generated by different nanoparticles may conflict with one another during the simultaneous detection of multiple biomarkers in the same sensor, and, as a result, the accuracy of the results may be reduced. This problem is solved using nanoparticles with different optical and electrical properties. Depending on the wavelength and color of the quantum dots, different biomarkers can be used, and those different biomarkers will create distinct signals that can help reduce interference between them. Furthermore, another challenge related to the development of nanoparticle surfaces is that each nanoparticle must be individually functionalized to stay bound to its specific biomarker during multiplex detection. Fortunately, nanoparticles can form biomolecular layers optimized to ensure high specificity while minimizing interference with other biomarkers in the indirect region. There is also a need for these surfaces to remain stable under complex biological environments, including those arising from biological specimen such as blood or feces, since these specimen contain various compounds that can interfere with these layers and reduce their ability to detect certain substances. In addition, the complexity of data processing in a multiplex assay is another challenge to overcome. To ensure accurate interpretation of information, a sophisticated algorithm needs to be developed to analyze several different signals from various biomarkers while simultaneously detecting several signals from the same biomarker. Additional challenges arise in low-resource environments that lack sophisticated data processing tools, which can prove particularly challenging.

Temperature and humidity strongly affect the nanobiosensor's lifetime, especially in tropical environments [214–217]. These factors can directly affect the chemical and physical stability of nanomaterials used in these sensors and biological molecules, such as antibodies or aptamers attached to them. In tropical environments, where high temperatures and humidity are common, these issues pose serious challenges to maintaining sensor performance and longevity. Theoretically, nanobiosensors should be usable for a certain period at ambient temperature. However, unwanted reactions and degradation of biomolecules occur in tropical environments with high temperatures (sometimes more than 40°C) and very high humidity. In many sensors, gold or silver nanoparticles are used as diagnostic labels. These particles may aggregate or undergo surface changes that deteriorate them at high temperatures. The efficiency of these systems can be reduced as a result of this. Moreover, antibodies and other biomolecules commonly used in nanobiosensors are sensitive to heat and moisture [58, 218]. This can reduce their performance by denaturing or determining whether they are no longer biologically active. Nanobiosensor lifetime and stability under these conditions have been improved through several strategies [219, 220]. Among these methods, one that is frequently used is the application of protective coatings onto nanoparticle materials and biomolecules so that they will be protected against the conditions of the environment. It can be made of stable polymers to protect the coating against temperature and humidity. Creating a protective layer over sensors prevents them from contacting any contaminating elements in the environment. In addition, these coatings can help maintain the internal humidity of the sensor and prevent rapid changes in the physical or chemical properties of the nanomaterials. Another solution is the development of nanobiosensors with more resistant biological compounds. Instead of antibodies sensitive to temperature and environmental conditions, aptamers or synthetic molecules with higher stability can be used. As biomolecules replace antibodies, aptamers are more resistant to different temperatures and pHs. As a result, they can be more efficient in tropical conditions. In addition, storing and transporting nanobiosensors under controlled environmental conditions can also increase their lifespan. Keeping these sensors in low-temperature or dry environments until their final use can prevent damage to biological materials and nanoparticles. For this purpose, advanced packaging systems that are resistant to humidity and heat can be helpful.

**Fig. 5:**
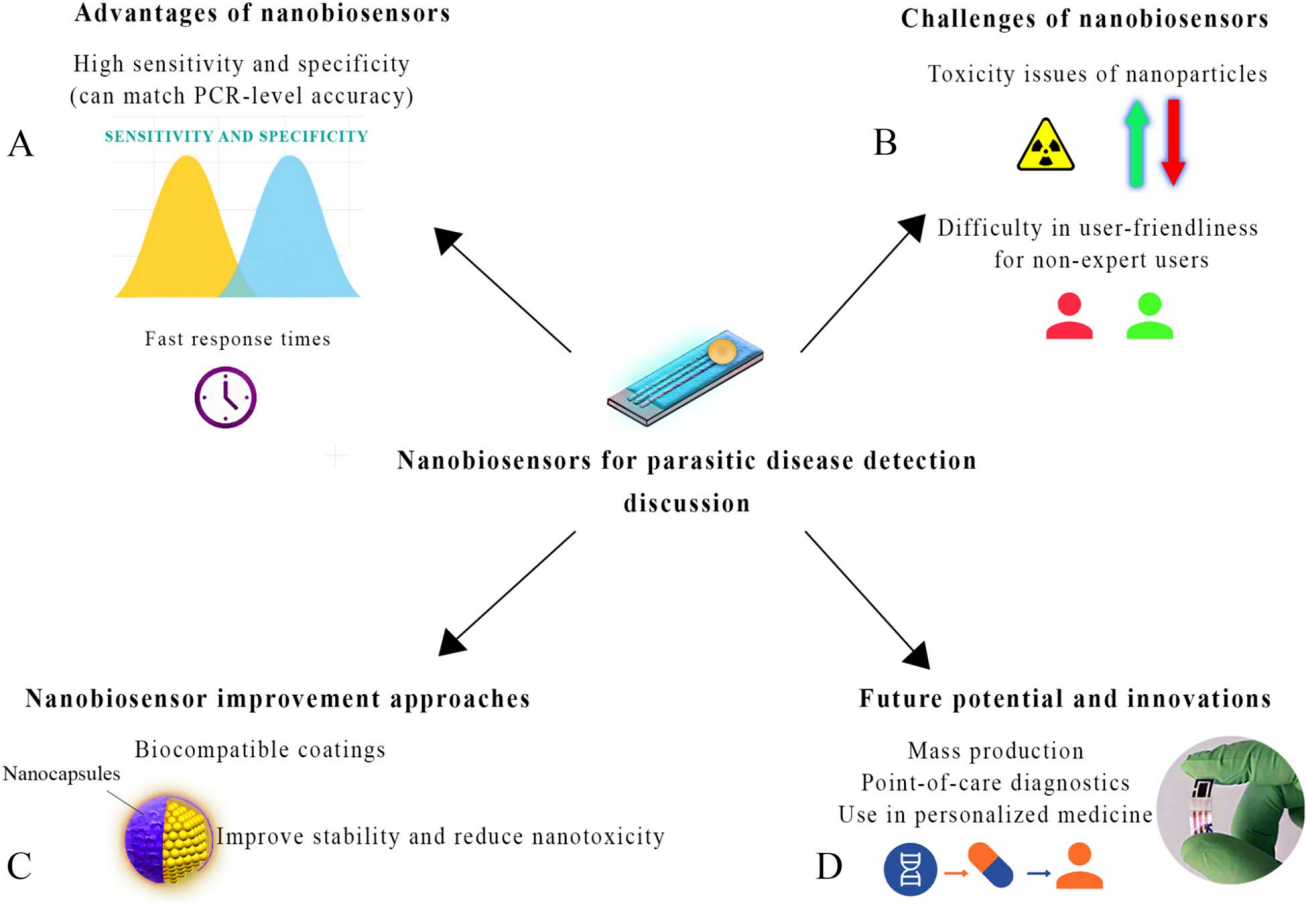
Advantages, challenges, and future directions of nanobiosensors in parasitic disease detection. **A** Key advantages include high sensitivity and specificity, comparable PCR-level accuracy, and fast response times. **B** Important challenges remain, such as nanoparticle toxicity and user-friendliness for non-experts. **C** To address these issues, improvements like biocompatible coatings (*e.g.*, nanocapsules) are proposed to enhance stability and reduce nanotoxicity. **D** Future innovations focus on mass production for point-of-care diagnostics and applications in personalized medicine

Despite their high potential, nanobiosensors pose essential ethical and legal issues in disease diagnosis [221–223]. These issues are raised concerning data privacy, informed patient consent, and possible disparities in access to this new technology. In diagnosing parasitic diseases, which are more prevalent in regions with limited resources and developing countries, attention to these aspects is critical to maintaining public trust and implementing responsible policies. Data privacy and patient personal information security are among the most significant ethical concerns. Nanobiosensors collect complex and accurate biological information from patients. This information includes genetic and biomarker data that can be used for medical diagnoses and beyond (such as predicting genetic predisposition to diseases) [224–226]. This raises concerns about the unauthorized use and storage of this data. Patient privacy risks are serious in environments without clear laws protecting personal data. Therefore, it is necessary to establish clear protocols for data protection and confidentiality principles in medical diagnostics before using nanobiosensors. Consent from patients is also one of the issues that must be addressed. Due to the complexity of the technology and its newness, it may be difficult for many patients to understand the purpose of using nanobiosensors, especially in areas of deprivation and a lack of access to health care. Nanobiosensors should be explained completely to the patient so they understand how they work, what kind of data is collected, and how they will use it [49, 227–229]. This process aims to provide people with accurate, helpful, and clear information in plain language to allow them to make decisions without having to be dictated to by someone else. In addition, where nanobiosensors are proposed to be used in a research project, informed consent should be provided by emphasizing the patient's right to withdraw from the study and ensuring their personal information is protected during the experiment. Another challenge is inequality in access to advanced technology. On the other hand, the lack of specific national and international laws to regulate nanotechnology and biotechnology in diagnosing diseases may fuel the misuse of these technologies. It is necessary to develop clear and precise regulations at the global level to determine legal frameworks for the safe and fair use of nanobiosensors (Fig. 5).

## Future prospects

Several steps need to be taken to make nanobiosensors available for diagnosing and treating parasitic diseases. The desired nanobiosensors are designed and developed based on diagnostic needs in the development and design stage [218, 230, 231]. Choosing suitable materials and optimizing the sensitivity and accuracy of nanobiosensors is very important. In the stage of preclinical tests, nanobiosensors are tested in laboratory conditions to evaluate their efficiency and accuracy in identifying parasites [232, 233]. In this stage of clinical trials, nanobiosensors are tested in clinical environments and on human specimen to check their efficiency and accuracy in natural conditions. After confirming the efficiency and accuracy of nanobiosensors, these products must obtain the necessary permits from legal authorities to be marketed [234, 235]. In this mass production and commercialization phase, nanobiosensors are mass-produced and marketed. Training users and proper distribution of products are also significant. After nanobiosensors are released to the market, their performance and efficiency should be continuously monitored and evaluated to ensure that the products work properly and potential problems are identified and fixed in time [236–239].

Nanobiosensors exhibit significant potential in revolutionizing the parasitic infections diagnosis. Utilizing nanobiosensors to detect parasites offers a promising opportunity to improve diagnostic skills. The primary factors to be considered while choosing nanobiosensors encompass their ability to selectively target parasite species and biomarkers and their capacity to detect antigens at low concentrations with a high level of sensitivity [240]. In addition, advancing innovative biomarkers and target-specific recognition elements will facilitate the precise identification of a diverse array of parasites. Carbon nanotubes and metallic nanoparticles can enhance the amplification of detection signals. The advancement of compact and portable diagnostic technologies allows for quick detection at the location, offering the potential for prompt intervention and treatment [241, 242]. Further research is essential to optimize nanobiosensors to enhance disease management and healthcare outcomes. Although nanobiosensors have great potential, their extensive implementation in detecting parasites encounters several challenges. A notable obstacle is the establishment of standardized assay procedures and validating sensor efficacy across diverse helminth and protozoan species. Ensuring accurate diagnosis and suitable treatment requires consistency and dependability in test results.

Even though nanobiosensors have the potential to revolutionize parasitic disease diagnosis due to their high sensitivity, rapidity, and capability of being used at the PoC, the transition from laboratory to clinical application of this technology is fraught with several challenges that need to be addressed in the future. Considering the safety and toxicity of the nanomaterials in animal and human models is vital to the project's success, since their accumulation in the body will likely cause long-term complications [243–245]. Furthermore, the stability and standardization of sensors are usually investigated in controlled laboratory environments to determine how well they perform in real-world situations (such as blood specimens with a complex composition or variable temperatures). At the same time, field conditions may impede the performance of the sensor. In addition, obtaining approval from reputable organizations such as the Food and Drug Administration (FDA) can be expensive and time-consuming, since it requires extensive validation studies on diverse populations and comparisons with gold standard testing methods, significantly increasing the cost and time of development [84, 246, 247]. In addition, it is essential to ensure that the scalability of production and cost-effectiveness of the technology are optimized to be cost-effective for health systems in low-income countries (where parasitic diseases are a significant problem). Researchers, clinicians, technology companies, and regulatory agencies must collaborate to resolve these challenges so that these technologies can be commercialized by conducting multicenter studies, developing integrated protocols, and investing in mass production to ensure that they are commercialized.

By integrating nanobiosensors with mobile platforms (such as smartphones), the Internet of Things (IoT), and the practice of telemedicine, nanobiosensor-based diagnostics could revolutionize the diagnosis and treatment of parasitic diseases in underserved areas by preventing the spread of parasites [188, 248]. It is thought that in the near future, nanobiosensors, based on nanoparticles and carbon nanotubes, will be able to process biological data in real time, which can be shown to doctors via the cloud by connecting to portable devices. There are a number of advantages to using this approach, including reducing the time from diagnosis to minutes, providing long-term monitoring of patients in areas with limited access to central laboratories, and improving patient outcomes [249]. For example, an IoT system equipped with nanobiosensors can be used to monitor the change in biomarkers, such as parasite antigens, in saliva or blood specimens and send an automated alert to health facilities when there is a change. As important as these advances are, there are still significant challenges to implementing these technologies, including the need for sustainable energy (long-life batteries), data security, and integration with existing infrastructure. By analyzing nanobiosensor data using machine learning algorithms, it is possible to increase the detection accuracy to levels equal to those achieved with conventional methods such as PCR.

By detecting multiple markers simultaneously with a technique known as spatial cellular indexing of transcriptomes and epitope sequences (CITE-seq), new methods can be used to increase the sensitivity of nanobiosensors for detecting biological specimens [250]. In addition, the development of perturbation-compatible deterministic barcoding in tissue (perturb-DBiT), which enables in situ clustered regularly interspaced short palindromic repeats (CRISPR) sequencing of parasites, may help optimize the design of nanobiosensors to deliver their detection more accurately to parasites via more precise targeting techniques [251]. There is also a potential role for "multimodal tri-omics mapping" methods that have been proposed to look at the interactions between genomic, transcriptomic, and proteomic data in dynamic tissues in the context of developing multifunctional nanobiosensors for parasite diagnosis. Furthermore, these technologies are capable of simultaneously detecting multiple pathogens, and they can also provide information about the mechanisms that are responsible for the interaction between parasites and their hosts. As a result, it is necessary to streamline the process and reduce costs in order for these methods to be applied to field conditions in low-income countries. Future studies need to focus on the development of modular nanobiosensors that can be connected to digital health networks and calibrated and repaired remotely as part of smart monitoring systems (Table 5).

**Table 5:** Key challenges hindering the widespread production of nanobiosensors for parasitic disease diagnosis, with actionable solutions and critical considerations. Focus areas include technical performance, cost, regulatory hurdles, and field adaptability

Challenge category	Specific challenge	Proposed solution	Key considerations
Technical	Low sensitivity for rare parasites	Multi-target nanobiosensors	Multiplexing capability
Technical	Signal interference in complex specimen	Advanced noise-reduction algorithms	Machine learning integration
Material Stability	Nanomaterial degradation in harsh conditions	Robust coatings (*e.g.*, PEG, silica)	Long-term field testing
Cost	High production costs	Scalable manufacturing (*e.g.*, roll-to-roll)	Affordable for low-resource settings
Regulatory	Lack of standardized validation protocols	Collaborative frameworks (FDA/WHO guidelines)	Multi-center clinical trials
Portability	Heavy readout devices	Smartphone-integrated sensors	Wireless connectivity (Bluetooth)
User-friendliness	Complex operation for non-experts	Automated specimen-to-result systems	Minimal training required
Biological	Non-specific binding	CRISPR-based target specificity	High-fidelity probes
Environmental	Temperature/humidity sensitivity	Thermostable nanomaterials (*e.g.*, DNA origami)	Field adaptability
Data Security	Privacy risks in IoT health monitoring	Blockchain-based encryption	Health insurance portability and accountability act, general data protection regulation systems
Sample Prep	Need for pre-processing	Direct detection in whole blood/bio specimen	Minimize steps
Scalability	Batch-to-batch variability	AI-driven quality control	Standardized fabrication

## Conclusions

Herein, we reviewed the application, advantages, challenges, and future prospects of nanobiosensors for early detection of globally important human parasitic diseases, i.e., malaria, leishmaniasis, CE, taeniasis, and schistosomiasis. We highlighted that nanobiosensors could potentially revolutionize parasitic infection detection and management through enhanced performance and accessibility through future nanotechnology and materials science advances. Considering the rapidly ongoing advancement of nanotechnology, it is expected that research and development in nanobiotechnology will increase, and nanobiosensors will be utilized to aid the detection, treatment, and management of parasitic infections by providing new avenues for early detection, improved treatment, and improved disease management in the future. Addressing the challenges associated with their implementation is essential for realizing their full impact on global health. Future research efforts should focus on overcoming these hurdles and harnessing the full potential of nanobiosensors in the fight against parasitic infections.

## Data Availability

No datasets were generated or analysed during the current study.

## Abbreviations

IHC: Immunohistochemistry
ELISA: Enzyme-linked immunosorbent assay
IB: Immunoblotting
PoC: Point of care
CE: Cystic echinococcosis
SEM: Scanning electron microscopy
TEM: Transmission electron microscopy
ZP: Zeta potential
EIS: Electrochemical impedance spectroscopy
SPR: Surface plasmon resonance
FRET: Fluorescence resonance energy transfer
Au: Gold electrode
*Pf*HRP2: *Plasmodium falciparum* Histidine-rich protein 2
HRP: Horseradish peroxidase
TMB: 3, 3′, 5, 5′Tetramethylbenzidine dihydrochloride
LOD: Limit of detection
FTIR: Fourier transform infrared spectroscopy
qPCR: Quantitative real-time PCR
TTAT: Therapeutic turnaround time
BSA: Bovine serum albumin
*p*LDH: *Plasmodium* Lactate dehydrogenase
GNP: Gold nanoparticle-enhanced platform
BREs: Biomolecular recognition elements
DA: Detection accuracy
CSPR: Conventional surface plasmon resonance
IFOM: Imaging figure of merit
CL: Cutaneous leishmaniasis/leishmaniosis
VL: Visceral leishmaniasis/leishmaniosis
LFA: Lateral-flow assay
CNS: Central nervous system
NiO: Nickel oxide
ITS2: Internal transcribed spacer 2
ThNT: Thiolated carbon nanotube
GO: Graphene oxide
GQD: Graphene quantum dots
CT: Computed tomography
FDA: Food and Drug Administration
LSPR: Localized surface plasmon resonance
CITE: Cellular indexing of transcriptomes and epitopes
SWA: Soluble worm antigens
AI: Artificial intelligence
perturb-DBiT: Perturbation-compatible deterministic barcoding in tissue
MBA: Mercaptobenzoic acid
CV: Cyclic voltammetry
PEG: Polyethylene glycol
IoT: Internet of Things
CRISPR: Clustered regularly interspaced short palindromic repeats
CITE-seq: Cellular indexing of transcriptomes and epitope sequences
